# Solvent-Free Approaches for the Processing of Scaffolds in Regenerative Medicine

**DOI:** 10.3390/polym12030533

**Published:** 2020-03-02

**Authors:** Víctor Santos-Rosales, Ana Iglesias-Mejuto, Carlos A. García-González

**Affiliations:** Department of Pharmacology, Pharmacy and Pharmaceutical Technology, I+D Farma group (GI-1645), Faculty of Pharmacy, Health Research Institute of Santiago de Compostela (IDIS), Agrupación Estratégica de Materiales (AeMAT), Universidade de Santiago de Compostela, E-15782 Santiago de Compostela, Spain; victor.santos.rosales@rai.usc.es (V.S.-R.); ana.iglesias.mejuto@rai.usc.es (A.I.-M.)

**Keywords:** regenerative medicine, scaffolds, gas foaming, 3D printing, supercritical foaming, melt molding, sintering

## Abstract

The regenerative medicine field is seeking novel strategies for the production of synthetic scaffolds that are able to promote the in vivo regeneration of a fully functional tissue. The choices of the scaffold formulation and the manufacturing method are crucial to determine the rate of success of the graft for the intended tissue regeneration process. On one hand, the incorporation of bioactive compounds such as growth factors and drugs in the scaffolds can efficiently guide and promote the spreading, differentiation, growth, and proliferation of cells as well as alleviate post-surgical complications such as foreign body responses and infections. On the other hand, the manufacturing method will determine the feasible morphological properties of the scaffolds and, in certain cases, it can compromise their biocompatibility. In the case of medicated scaffolds, the manufacturing method has also a key effect in the incorporation yield and retained activity of the loaded bioactive agents. In this work, solvent-free methods for scaffolds production, i.e., technological approaches leading to the processing of the porous material with no use of solvents, are presented as advantageous solutions for the processing of medicated scaffolds in terms of efficiency and versatility. The principles of these solvent-free technologies (melt molding, 3D printing by fused deposition modeling, sintering of solid microspheres, gas foaming, and compressed CO_2_ and supercritical CO_2_-assisted foaming), a critical discussion of advantages and limitations, as well as selected examples for regenerative medicine purposes are herein presented.

## 1. Introduction

The change in the demographic paradigm due to the aging of the population and new social habits taking place in the majority of the developing countries results in an increase of osteochondral fractures and diseases that affect the mobility, autonomy, and quality of life of thousands of million people worldwide [1,2]. For critical bone defects, bone grafts from the same patient or from donors is the routine clinical practice, although side effects and post-surgical difficulties may also take place (slow or deficient bone recovery, donor bone site morbidity, limited availability, rejection, risk of disease transmission, and low osseointegration, among others) [3]. The regenerative medicine field offers the possibility of revisiting the current options for the treatment of osteochondral pathologies.

The clinical complications associated with biological grafts coupled with their limited availability have boosted the research on the design and development of synthetic polymeric grafts acting as 3D scaffolds that are able to contribute to tissue regeneration and guide the growth of the tissue in the region to be repaired. Despite intensive research being undertaken on the topic, the development of a robust production method leading to effective synthetic bone scaffolds has not been solved yet.

The efficient regeneration of a fully functional tissue assisted by the presence of a polymeric scaffold is crucially dependent on the scaffold design (composition, morphology, mechanical, and biological properties) and the processing method used [4]. The portfolio of synthetic polymers that are susceptible to being used as components in a scaffold is severely restricted by their biocompatibility, biodegradation rate, and metabolism [5,6]. On the other hand, the incorporation of bioactive agents (e.g., growth factors, drugs) during the scaffold manufacturing is of particular interest to improve the environment–scaffold interactions, accelerating its integration and ensuring precise tissue regeneration [7]. The resulting scaffolds, the so-called medicated scaffolds, represent a step-forward approach of its clinical use, since the tunable spatio-temporal release of the therapeutic agents could be matched according to the tissue requirements after its implantation, also alleviating the patient health status (immunomodulatory [8,9,10] or anti-microbial effects [11]). Regarding scaffold manufacturing, many processing methods are regarded as inefficient and expensive techniques due to the number of processing steps used, the need for exhaustive purifications and, in the particular case of medicated scaffolds, the low incorporation yield and limited spatial control of the loaded bioactive agents [12].

Processing technologies to obtain scaffolds should be reproducible, have few steps and easy scale-up, and be cost-competitive for the sake of the quality of the material, health safety, and economy of the process. Several methods have been developed for the processing of polymeric scaffolds [6,13] comprising two main groups: (1) methods using solvents to solubilize the polymer or to incorporate binders, and (2) methods processing the polymer under a viscous behavior (usually above the glass transition or melting point of the polymers). These processing methods have differing success rates regarding the control of the scaffold performance. The optimum technological solution is the fabrication of the scaffold in one single step (i.e., no post-processing) and in the absence of organic solvents that could compromise the biocompatibility of the scaffold and with customized size and morphology.

In the case of medicated scaffolds, high loading yields of the bioactive agents in the material with predictable and therapeutically relevant release profiles after implantation are critical parameters to select the processing approach due to their high cost. In general, the processing of scaffolds in the presence of solvents hampers the control of the spatial distribution of the bioactive agent in the porous structure of the scaffolds upon processing and under storage. In case of leaching or solvent extraction steps, dramatic removal of the bioactive agent along with the presence of residual porogens or toxic organic solvents are among the drawbacks that might take place in the end material. Consequently, the processing technologies operating in the absence of any solvent during the assembly of the 3D scaffolds arise as an auspicious strategy to overcome the abovementioned problems with medicated synthetic grafts.

Melt molding (compression, injection, and extrusion molding), 3D printing by fused deposition modeling, the sintering of solid particles (heat, compressed CO_2_, and selective laser sintering), gas foaming, and compressed/supercritical CO_2_ foaming are the main solvent-free strategies for the processing of scaffolds. The fundamentals and the main results obtained for these technologies so far in regenerative medicine as well as the advantages and disadvantages for each one will be discussed in the following sections. A particular focus of this review will be devoted to the potential use of these solvent-free strategies for the processing of medicated scaffolds. To best of our knowledge, this work represents the first attempt to classify and assemble the technological portfolio of solvent-free processing methods for scaffolds manufacturing.

## 2. Melt Molding

**Figure 1:**
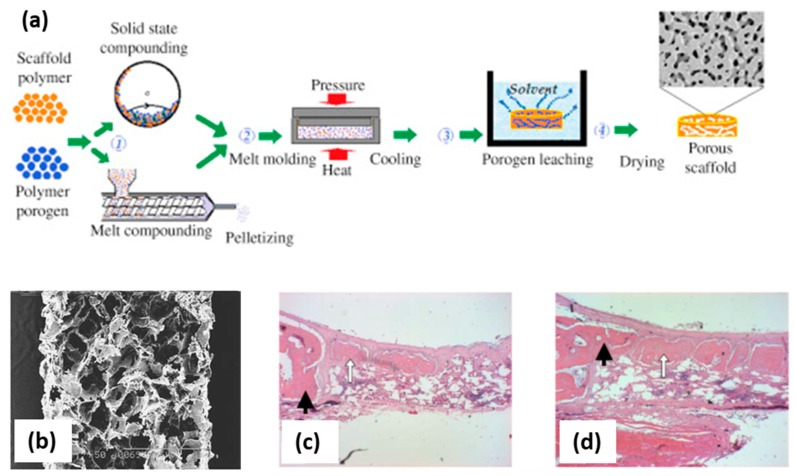
(**a**) Sketch of the compression molding technology combined with porogen leaching to obtain a porous scaffold [14]. Briefly, a polymer in the powdered form is mixed with a porogen to form a composite that is granulated and melt molded upon pressure and heat. Then, the obtained material is placed in a solvent bath for porogen leaching followed by a drying process to get a dry porous scaffold. (**b**) Cross-section of poly(lactic-co-glycolic acid)/polyvinyl alcohol (PLGA/PVA) scaffolds obtained by melt molding (scale bar: 1 mm); histological sections of these scaffolds after (**c**) 3 and (**d**) 6 weeks implantation show the formation of new bone (white arrows) in the host bone (black arrows). Figure 1a reprinted from Functional 3D Tissue Engineering Scaffolds, Rula M. Allaf, Chapter 4. Melt-molding technologies for 3D scaffold engineering, 75–100, Copyright 2018, with permission from Elsevier; (**b**–**d**) reprinted form Biomaterials, 24, Se Heang Oh, Soung Gon Kang, Eun Seok Kim, Sang Ho Cho, Jin Ho Lee, Fabrication and characterization of hydrophilic poly(lactic-coglycolic acid)/poly(vinyl alcohol) blend cell scaffolds by melt-molding particulate-leaching method, 4011-4021, Copyright 2003, with permission from Elsevier.

Melt molding technology applies heat inputs for the processing of thermoplastic polymers, which are usually above the glass transition for amorphous polymers and above the melting point for semi-crystalline or crystalline polymers [14]. The heating energy demands of this technology will depend on the processing temperature needed as well as the specific heat capacity of the polymeric matrix. Compression molding, injection molding, and extrusion molding are the three main variants of the melt molding approach depending on the polymer heating operation mode and the shaping element used (die or mold) (Figure 1a). Materials with complex external geometries can be obtained by modifying the morphology of the mold or die used. This technology is scalable, reproducible, and cost-effective, rendering it the most commonly used for the commercial processing of polymeric fixation elements in orthopedics. Moreover, scaffolds for regenerative medicine processed using this technology were obtained with open-cell structures, high porosity, and suitable pore sizes (Figure 1b) [15,16]. Namely, scaffolds of poly(lactic-co-glycolic acid) (PLGA) with polyvinyl alcohol (PVA) processed using this technique were evaluated in vivo (skull non-critical 10 mm diameter defect in New Zealand white rabbit model) showing good biocompatibility with bone ingrowth and the formation of new mature bone 6 weeks after implantation (Figure 1c,d) [15]. Nevertheless, this technology has severe limitations for porous materials (e.g., in scaffolds for regenerative medicine) and a combination with pore-generating techniques such as particulate leaching (by porogen particles removal) or phase separation (by selective polymer dissolution) is needed to favor pore formation and to refine the porous structure (Figure 1a) [17,18,19]. The use of this post-processing step neglects the above-mentioned advantages of a solvent-free approach for medicated scaffolds. Moreover, quality problems can take place by the melt molding of biopolymers due to the poor thermal conductivity of polymers coupled to their limited thermal stability [14]. Local or regional hotspots leading to the partial thermal degradation of the polymer and slow post-molding solidification step of the material taking place upon processing are straightforward consequences of the thermal behavior of thermoplastic polymers.

## 3. D-Printing by Fused Deposition Modeling (FDM)

**Figure 2:**
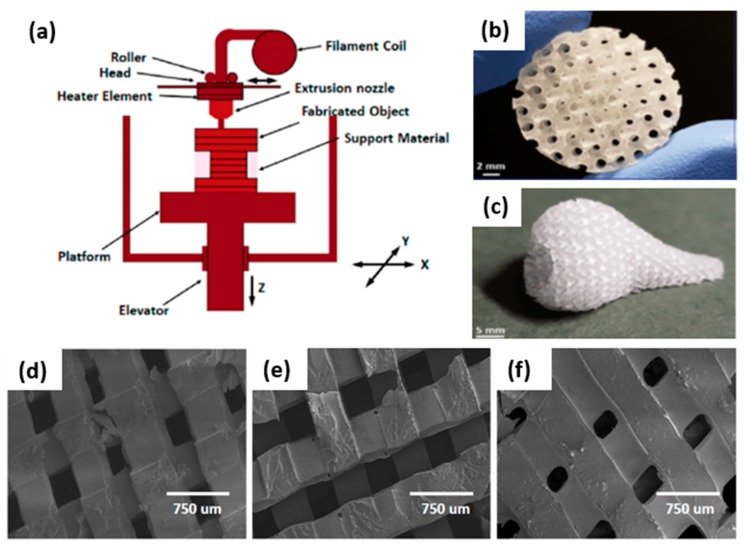
Solvent-free 3D scaffolds obtained by the fused deposition modeling technique: (**a**) Thermoplastic materials are molten into a liquid state in a liquefier head. Then, compounds are deposited through a nozzle that obeys a computer-aided design (CAD) file and generate 3D parts in a layer-by-layer fashion [23]. (**b**) Polydimethylsiloxane (PDMS) scaffold fabricated with complex geometries and gradient porous structures, including uniform and graded distributions with different pore shapes. (**c**) Porous nose fabricated with a fused deposition modeling (FDM) printing approach and showing the potential to generate structures with complex architectures to achieve human organ shapes [26]. (**d**–**f**) SEM images of PLA templated poly-(esterurethane) (PUR) scaffolds fabricated by fused deposition modeling with tunable substrate modulus (5, 24 and 266 MPa) [27]. (**b**,**c**) reprinted from Acta Biomaterialia, 96, Montazerian, H.; Mohamed, M.G.A.; Montazeri, M.M.; Kheiri, S.; Milani, A.S.; Kim, K.; Hoorfar, M, Permeability and mechanical properties of gradient porous PDMS scaffolds fabricated by 3D-printed sacrificial templates designed with minimal surfaces, 149-160,2019, with permission from Elsevier. (**d**–**f**) reprinted from Biomaterials, 73, Guo, R.; Merkel, A.R.; Sterling, J.A.; Davidson, J.M.; Guelcher, S.A., Substrate modulus of 3D-printed scaffolds regulates the regenerative response in subcutaneous implants through the macrophage phenotype and Wnt signaling, 85–95, 2015, with permission from Elsevier.

Fused deposition modeling (FDM) is a mature additive manufacturing technology with broad application range and a high throughput/cost ratio [20,21]. FDM technology is simple, flexible, and does not usually require post-printing processes unless support material removal or polishing are needed [21,22]. Thermoplastic compounds in filament or powder forms are used as starting material and are molten and extruded through a high-temperature nozzle onto an *x*-*y*-*z* platform (Figure 2a). The nozzle moves in the *x*- and *y*-direction and is computer controlled. The fused material solidifies and deposits layer by layer onto a built platform that can move in the *z*-direction to complete the 3D design dictated by a model in a computer-aided design (CAD) file [21,23]. After the first layer is completely extruded from the nozzle, the print bed lowers a fixed distance in the *z*-axis corresponding with the layer thickness. Then, the next layer can be printed over the original one [24]. Each layer is fused and bonded with the layer below [20,23]. The formed layer must be kept at temperatures below the materials’ solidification peak to ensure good interlayer adhesion [25]. The final scaffold architecture is determined by parameters such as the nozzle diameter, deposition speed, deposition angle, layer thickness, or space between filaments in the same layer [21].

Synthetic polymers are the most widely used materials to form scaffolds for cartilage/bone treatment by FDM due to their tunable mechanical properties, degradability, and biocompatibility. These polymers exhibit controllable degradation properties and can be fabricated to desired shapes. In the case of synthetic polymers, the most used include polyglycolic acid, polylactic acid, polylactic-co-glycolic acid, polycaprolactone, and polyvinyl alcohol [28].

For regenerative medicine, FDM can be used for the processing of scaffolds with highly controllable porosity (Figure 2b) and good mechanical properties [29,30]. Furthermore, FDM can be used to obtain combinations of specific scaffold geometries and mechanical properties while preserving cellular behavior. Additionally, the flexibility of operation allows the manufacturing of individual batches of scaffolds personalized to a specific defect obtained from a patient by X-ray computed tomography (CT) or magnetic resonance imaging (MRI) techniques [25]. Using this Computer-Aided Tissue Engineering technique, unique functional scaffold pieces of personalized shape can be printed (Figure 2c), addressing osteochondral defects or designing scaffolds for complex-shaped human organs [26,31,32]. This technique plays an important role in the manufacturing of porous tissue scaffolds for the regeneration of tissues with an appropriate shape and size so that cells can penetrate on it when nutrients are provided. An ideal scaffold consists of a porous structure through which cells can proliferate with the proper supply of nutrients. To design and develop tissue porous scaffolds, parameters such as the porosity, mechanical strength, shape, and size of original tissue are provided on a 3D stereolithographic (STL) file to be developed through the computer-aided design (CAD) file generated from medical images. Using this process, 3D scaffolds are obtained for regenerate tissue or organs of specific shape and size from anatomical structural data provided by medical images from X-ray, ultrasound, MRI, or CT scans. This method comprises medical image acquisition and processing to convert the scan image into a 3D model, computer-aided designing to modify the images to adjust it per the requirement of the person to generate the patient-specific 3D CAD model, and finally, an additive manufacturing process dictated by the STL file [33].

The major limitation for FDM use is the high processing temperature required, which may degrade some drugs and excipients used in the scaffold formulations, limit the incorporation of biological molecules during extrusion, and usually compromise cell viability [22]. In addition, the rough surfaces obtained, the necessity of a support in some cases that should be removed, and the limited horizontal (100–150 µm) and vertical resolution (*ca.* 100 µm minimum layer thickness) of FDM printers in comparison with other 3D-printing methods are other disadvantages of this process [22,26,27,34]. Namely, FDM resolution is limited by the raw material properties, equipment specifications, and dimensions of extruded filaments [21,24,35].

**Figure 3:**
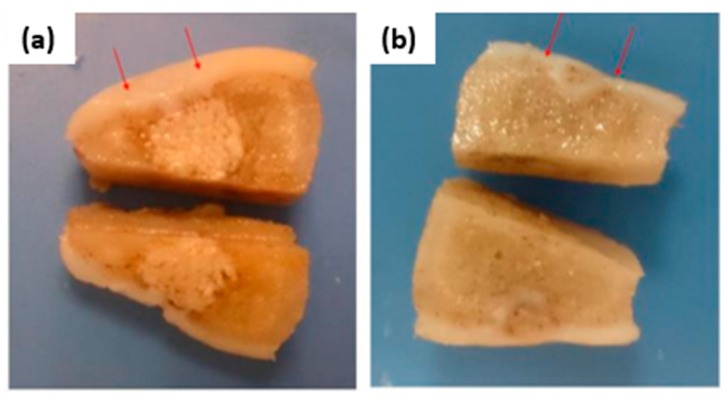
(**a**) Knee joint of the experimental group (biphasic composite scaffold implantation): The defect was filled with hyaline cartilage; subchondral bone was repaired, and non-degraded scaffold in the deep part of the femur condyle as well as bone tissue growth in the pores of the scaffold was noted. (**b**) Knee joint of the control group (without implants): The defect site was filled with hypertrophic cartilage-like tissue and invasion into subchondral bone area was observed. Reproduced from reference [36].

Porous scaffolds designed on minimal surface architectures and fabricated through a mold printing approach with an FDM 3D printer can stimulate regenerative wound healing and contribute to tissue mechanical properties as well. Nowadays, both facts drive interest in scaffolds for tissue regenerative applications such as the treatment of large cutaneous defects [26,27]. For example, 3D poly-(esterurethane) (PUR) scaffolds were fabricated with tunable substrate modulus (5, 24, and 266 MPa) (Figure 2d–f). It was demonstrated that the elastic modulus of the scaffolds influences scar formation both through the organization of fibroblasts infiltrating the wound bed and through the abundance of the extracellular matrix they deposit. Regenerative response, cellular infiltration, collagen deposition, and angiogenesis were maximized for wounds treated with scaffolds having a substrate modulus similar to that of collagen fibers (24 MPa) [27]. As another example, biomimetic scaffolds of poly(ε-caprolactone)/hydroxyapatite and glycidyl-methacrylate-modified hyaluronic acid were designed to be implanted in the knee joint of minipigs for healing osteochondral defects. The injured articular cartilage layer and subchondral bone were both regenerated and regrown. In addition, defect filling, integration to the surrounding host cartilage, and macroscopic appearance were better than those of the control group (empty defect) (Figure 3a,b) [36].

Finally, FDM-strategies to load drugs in 3D-printed scaffolds were developed as a way of customizing the drug release patterns for regenerative medicine aims [37,38]. One approach consisted of soaking PLA filaments into a drug solution before printing the scaffold, and another consisted of printing the scaffolds and then soaking them in the drug solution [39]. The combination of both methods offers the chance of creating concentration gradients of drugs with distinct release profiles in the same scaffold (dually loaded scaffolds). Scaffolds showed a mechanical behavior similar to that of human cancellous bone. The fast release profile of the drug was found on scaffolds loaded after printing, whereas scaffolds printed using drug-loaded PLA filaments could induce faster and prolonged osteogenic differentiation.

## 4. Sintering of Solid Microspheres

The use of microspheres (particle diameter ranging from 1 to 1000 μm) encapsulating bioactive compounds have been widely exploited for drug delivery applications due to their ability to provide controlled spatiotemporal drug release [40]. Microspheres can be assembled together to form an integrated porous 3D structure by applying a sintering treatment. The fused microspheres can act as the single or major component of the formed scaffold and allow the manufacturing of specifically designed shapes including bioactive molecules or cells, depending on the sintering method [41,42]. Microsphere-based scaffolds have undergone a huge development due to their inherent excellent mechanical behavior coupled with their controlled drug release with promising outcomes in vivo for bone and cartilage regeneration [43,44]. The production of microspheres for their use as “building blocks” in scaffolds commonly requires the use of organic solvents; the emulsion-solvent extraction method is one of the most widely used [45,46]. Nevertheless, the following subsections are only focused on the solvent-free sintering approaches for the assembling of these engineered microspheres as 3D scaffolds.

### 4.1. Heat Sintering Method

**Figure 4:**
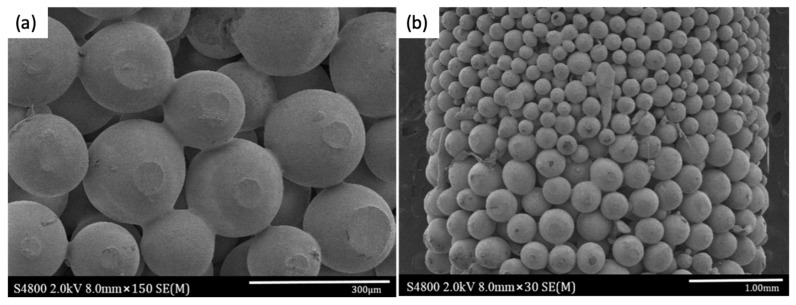
(**a**) Fused poly (D, L-Lactide) (PDLA) microspheres coated with TiO_2_ nanoparticles at 90 °C for 45 min and (**b**) detail of the pore size gradient of the scaffold obtained by the heat sintering method [49]. Figure reprinted from Nanomedicine: Nanotechnology, Biology and Medicine, 13, Morteza Rasoulianboroujeni, Mostafa Yazdimamaghani, Payam Khoshkenar, Venkata Raveendra Pothineni, Kwang Min Kim, Teresa A. Murray, Jayakumar Rajadas, David K. Mills, Daryoosh Vashaee, Keyvan Moharamzadeh, Lobat Tayebi, From solvent-free microspheres to bioactive gradient scaffolds, 1157–1169, 2017, with permission from Elsevier.

This method relies on the packing of microspheres according to the desired ending-scaffold structure and subjects them to a temperature (above the glass transition temperature, T_g_) for a certain period of time in order to induce the coalescence of particles by melting its surface, thus inducing new joints among them [47]. The degree of fusion depends on the sintering temperature and time, varying from the formation of slight new bonding to the pore occlusion and corresponding loss of interconnected porosity when over-sintering conditions are achieved. This method is simple, low cost, highly efficient, and compatible with the manufacturing of scaffolds with complex architectures such as pore size gradients (Figure 4) [48,49].

**Figure 5:**
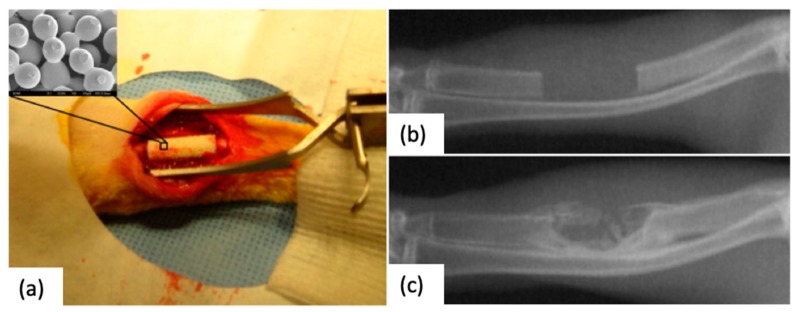
(**a**) Implantation of a sintered microsphere-based scaffold in a 15 mm induced ulna defect. (**b**) Appearance of the defect immediately after the surgical procedure, (**c**) radiograph on the defect site after 12-week post-operation with clearly new bone formation [43]. Figure reprinted from Acta Biomaterialia, 6, Tao Jiang, Syam P. Nukavarapu, Meng Deng, Ehsan Jabbarzadeh, Michelle D. Kofron, Stephen B. Doty, Wafa I. Abdel-Fattah, Cato T. Laurenci, Chitosan–poly(lactide-co-glycolide) microsphere-based scaffolds for bone tissue engineering: In vitro degradation and in vivo bone regeneration studies, 3457–3470, 2010, with permission from Elsevier.

The incorporation of bioactive agents during this scaffold processing method is not trivial, and the thermal stability of the drug of interest must be considered [50], although the use of inorganic compounds such as bioactive glasses [51] is feasible. On the other hand, the immobilization of proteins in chitosan/poly(lactide-co-glycolide) microsphere-based scaffolds by a post-processing step has been already demonstrated with enhanced in vivo performance regarding early bone formation [43,52] (Figure 5). In addition, scaffolds obtained by this technology can be used as cell substrates for 3D culture since they mimic the native extracellular matrix (ECM), thus being of interest for ex vivo tissue synthesis [53,54,55].

### 4.2. Compressed CO_2_ Sintering Method

This method is based on the plasticizer effect of CO_2_, being able to liquefy many polymers below their glass transition temperatures (T_g_) and melting points (T_m_) [56]. The extent of the sintering mainly depends on the operating pressure, exposure time, and working temperature, as these processing parameters determine the CO_2_ sorption on the polymeric particles [4,46]. The plasticization effect is meant to be achieved only at the surface to maintain the microspheres’ shape during the process while sintering adjacent particles. This approach avoids the use of organic solvents and high temperatures, maintaining the shape-specific scaffold manufacture. Compared to other sintering methods, the main advantage relies on the ability to fabricate cell-seeded scaffolds in a single-step process [57,58]. Nevertheless, the well-known sterilization ability of CO_2_ constrains the cell loading to scaffolds processed at high pressures or long expositions times [59]. Despite the advantages of the method, a paucity of research has been published and mainly focused on skeletal and cartilage tissue regeneration [60,61,62].

### 4.3. Selective Laser Sintering (SLS)

Selective laser sintering is an additive manufacturing technique for the production of 3D structures in a layer-by-layer manner based on a predefined computer-aided design (CAD). SLS uses a CO_2_ laser to induce a local increase of temperature above the T_g_ of the used polymer, leading to the coalescence of the adjacent particles. The process usually occurs under an inert atmosphere and it implies the following steps: (1) formation of a powder bed, (2) scanning by the laser beam to fuse the powder on the selected area, and (3) repetition of the former stages until the complete formation of the product. The resulting scaffold mechanical and morphological properties are determined by the amount of energy supplied to the polymeric particles, being a relationship between the laser power, the scan spacing, and beam speed [63,64]. SLS is a single-step process that offers products with higher resolution due to the laser precision (sub-millimeter region [65]), compared to other 3D-printing solvent-free processes such as FDM, as well as the ability to manufacture protruding regions without supporting materials. The main advantages of SLS for tissue engineering applications are related to the processability of a wide range of biomaterials, including ceramics, thermoplastic polymers [66,67,68,69], and composites [70,71,72,73,74,75,76,77,78], and the high customization degree. Namely, PCL sintered patient-specific external airway splints were implanted in infant patients suffering a life-threatening cardiopulmonary disease (tracheobronchomalacia), to prevent the collapse of the airways during respiration. In addition, scaffolds obtained from this technology inherently present rough surfaces that are required for the cellular attachment phenomena [79,80].

**Figure 6:**
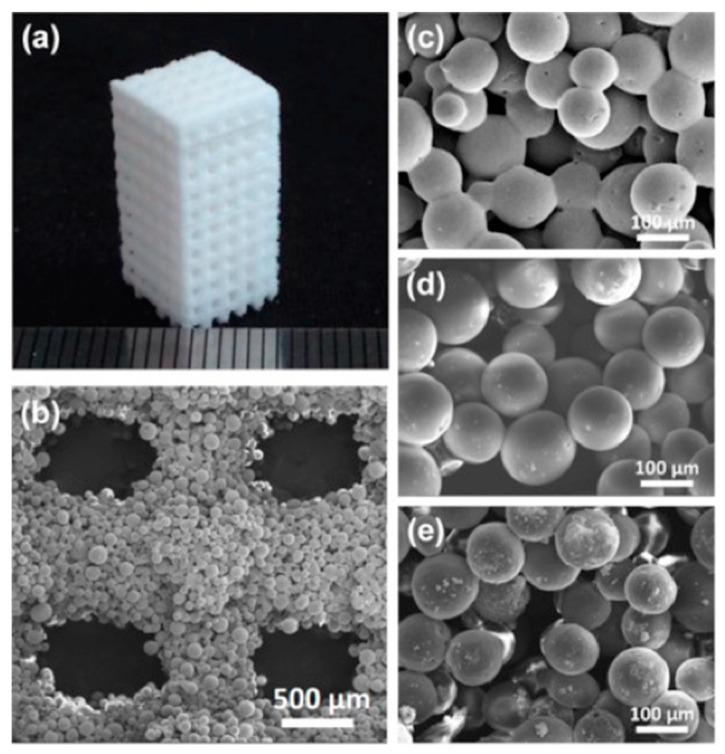
(**a**) Sintered cuboid porous scaffold with 3D orthogonal periodic porous architectures (length/width 8.8 mm, height 18.4 mm, pore size 800 µm). (**b**) SEM image of the pore morphology obtained in a 10% HA/PCL scaffold. (**c**–**e**) SEM images of the sintered PCL (**c**) and composite microspheres with increasing content of HA, (**d**) 10% HA/PCL, and (**e**) 20% HA/PCL. Figure reprinted from *Colloids and Surfaces B: Biointerfaces*, 135, Du, Y., Liu, H., Shuang, J., Wang, J., Ma, J., and Zhang, S., 81–89, 2015.

Nevertheless, standard SLS machines have remained at industrial scale and thus require large quantities of material in the adequate powder form, making the process very expensive. Modifications of commercial SLS printers that are able to produce porous scaffolds for tissue engineering applications and using low quantities of biomaterials are being proposed to reduce the economic burden [81,82,83]. Scaffolds formed by SLS can also be endowed with several biofunctionalities by incorporating bioactive agents [84]. These medicated scaffolds had a fully interconnected porous architecture (pore sizes ca. 200 µm) displaying a suitable mechanical and biological performance matching the criteria for their application as bone graft substitutes [85]. In addition, the sintering of PCL and hydroxyapatite (HA)/PCL composite microspheres as building blocks instead or raw powders enhanced the micron-scale porosity successfully, inducing the in vivo osteochondral regeneration using a rabbit model [86,87] (Figure 6).

## 5. Gas Foaming

The principle of this technique is the generation of pores in a polymeric matrix through a nucleation-growth mechanism of gas bubbles that after venting results in a macroporous material. The solvent-free version of this technique consists of three steps: (1) **Dispersion of a porogen in a polymeric matrix**. This porogen can be either a *chemical blowing agent*, i.e., a substance that is able to decompose into an inert gas by a chemical reaction (e.g., sodium bicarbonate) or by thermal decomposition (ammonium carbonate), or a *physical blowing agent*, i.e., an inert gas (nitrogen, argon, or carbon dioxide) insufflated or a volatile liquid (pentane) absorbed in the polymeric bulk. (2) **Pore formation through the porogen removal**. Gas bubbles are generated in this step following a nucleation-growth mechanism that results in pore formation after gas release. (3) **Rapid solidification of the polymeric matrix**. The temperature is lowered in a narrow timeframe to allow the vitrification of the material by freezing in order to avoid the destabilization of the resulting foam. Materials obtained by this technique are expanded polymeric foams with closed-cell or open-cell structures commonly used in the building sector. Gas foaming can be adapted for regenerative medicine purposes by means of the correct polymer–porogen match with biocompatible raw materials and degradation products and the processing of open-cell structures with suitable pore sizes and interconnectivity for cell growth and extracellular matrix secretion.

**Figure 7:**
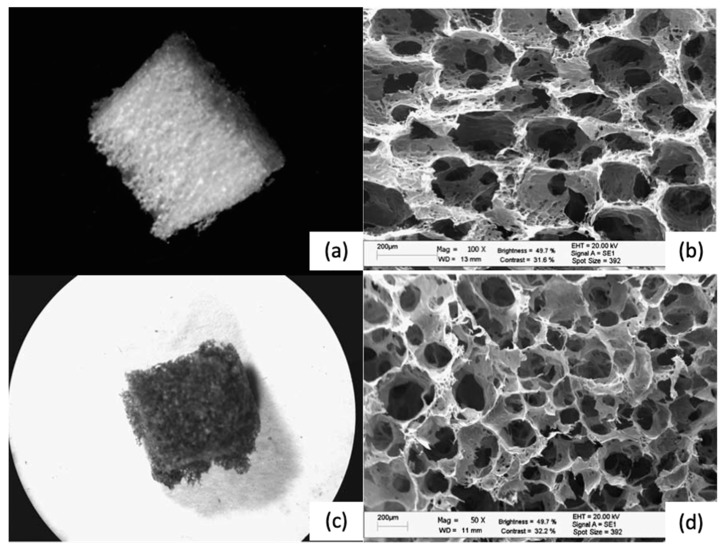
(**a**,**b**) Alginate scaffolds after the foam stabilization treatment (freeze-drying and cross-linking). (**c**,**d**) Chitosan scaffolds obtained by the same approach [90]. Reproduced from [90] with permission from The Royal Society of Chemistry.

Gas foaming based on chemical reactions exploits the generation of hydrophobic gas bubbles in an aqueous polymeric solution, thus being only available for hydrophilic biopolymers (e.g., gelatin or alginate) [88,89]. This approach usually results in wide pore size distributions, leading to anisotropic environments that negatively influence cell migration. Moreover, long processing times for complete porogen removal may be required. Conversely, the insufflation of an inert gas produce more homogeneous foams, as the volume of gas can be finely controlled. The capability of this approach has been enlightened by the processing of many polysaccharides (and potentially for all water soluble polymers) followed by fast freezing and lyophilization of the formed foam, obtaining porous scaffolds with interesting morphologies [90,91] (Figure 7), after a cross-linking post-treatment.

The gas foaming process is compatible with hydrophobic and hydrophilic polymeric matrices and is usually performed under mild temperatures. These properties, coupled to the fact that it is a solvent-free technology, result in a process that is suitable for the incorporation of bioactive agents in medicated scaffolds. Moreover, the foaming process can be carried out using molds that will cast the foam to a shape fitting an anatomical defect [92]. This technology may have limitations in the control of the pore size, pore interconnectivity, and spatial homogeneity of the material depending on the rheological properties of the liquefied polymer during the foaming [93].

## 6. Compressed CO_2_ and Supercritical CO_2_-Assisted Foaming

**Figure 8:**
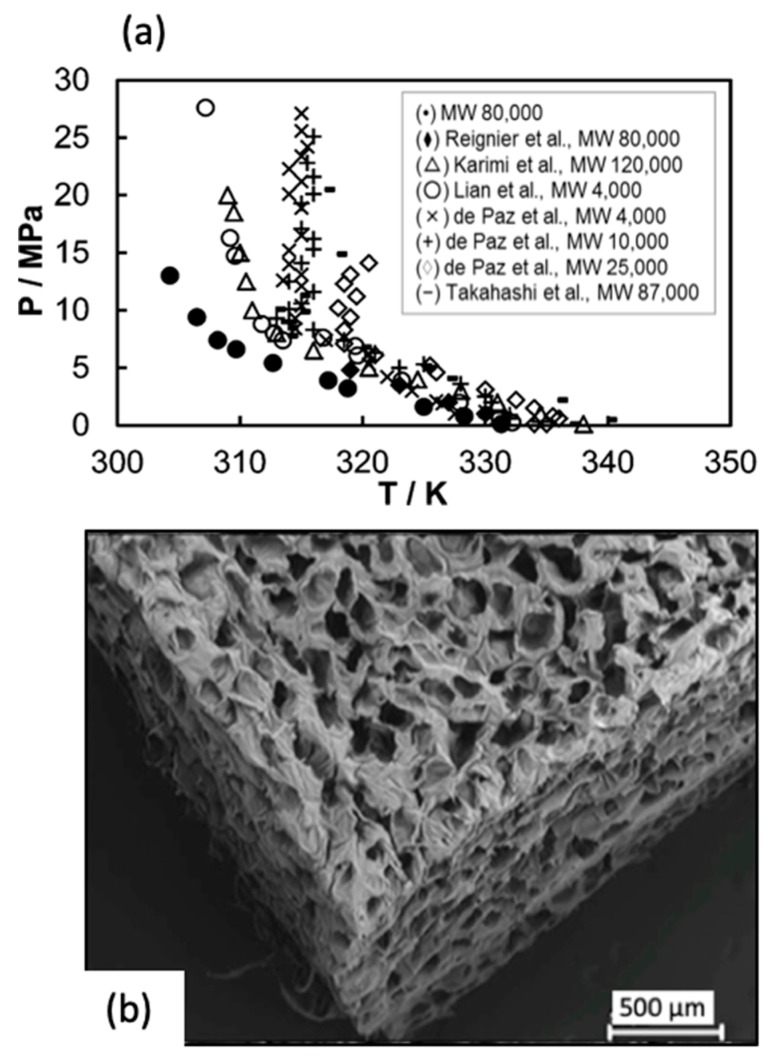
(**a**) Solid–liquid transition for PCL of different molecular weights (MW) in the presence of CO_2_ under certain pressure (y axis) and temperature (x axis) [95]. (**b**) Micrograph of PCL scaffold obtained by supercritical foaming (37 °C, 100 bar, 30 min soaking time) [96]. Figure 7a reprinted with permission from (Industrial and Engineering Chemistry Research 2013. 52(44), 15594-15601). Copyright (2013) American Chemical Society. Figure 7b reprinted from Carbohydrate Polymers, 142, Luis Diaz-Gomez, Angel Concheiro, Carmen Alvarez-Lorenzo, Carlos A. García-González, Growth factors delivery from hybrid PCL-starch scaffolds processed using supercritical fluid technology, 282–292, 2016, with permission from Elsevier.

Compared to other physical foaming agents, carbon dioxide presents unique advantages mainly related to its safety properties (low toxicity and flammability), recyclability, and sustainability being considered a green technology [94]. The morphology of the polymer (crystalline or amorphous) determines the free volume available for the CO_2_ to absorb in the polymers, affecting both the solubility and diffusivity values in the matrix [95]. However, the pressure/temperature-tunable physicochemical properties of CO_2_ under supercritical conditions permit modulating the polymer–fluid interactions. The compressed and supercritical CO_2_ foaming approach is based on the CO_2_ sorption and dissolution in the polymeric matrix under a targeted pressure and subsequent decompression to induce the polymer expansion. The plasticizer effect of CO_2_ induces a drop of the T_g_ and/or T_m_ of the polymer that is usually dependent on the operating pressure (Figure 8a). Therefore, lower working temperatures are required to process polymeric scaffolds using this technique than for other thermal processing methods with the pure polymer at atmospheric pressure.

**Figure 9:**
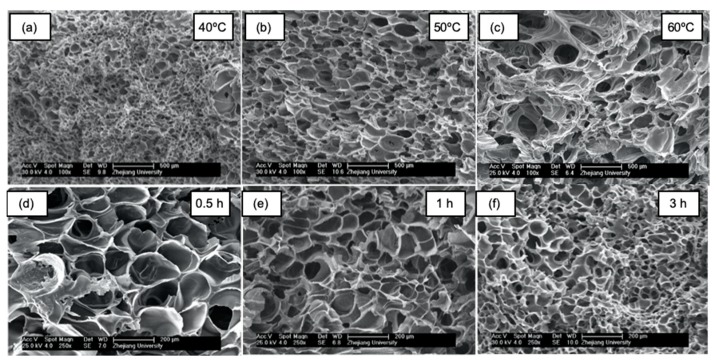
Morphological analysis by SEM imaging of PCL scaffolds processed by supercritical CO_2_ foaming at 10 MPa. (**a**–**c**) At a given soaking time (2 h), increasing the working temperatures induced the formation of larger pores. (**d**–**f**) Conversely, for a specific foaming temperature (40 °C), longer soaking times render scaffolds displaying smaller pores with greater pore numbers. Figure reprinted from The Journal of Supercritical Fluids, 117, Chen, C.-X., Liu, Q.-Q., Xin, X., Guan, Y.-X., and Yao, S.-J., Pore formation of poly(ε-caprolactone) scaffolds with melting point reduction in supercritical CO2 foaming, 279–288, 2016, with permission from Elsevier.

The operating temperature depletion associated with compressed CO_2_ foaming makes possible the incorporation of thermolabile bioactive compounds such as drugs [97,98,99,100,101,102] and proteins (e.g., growth factors, enzymes) [96,103,104,105,106,107], among others. Upon system depressurization, CO_2_ exits the polymeric matrix, leading to a scaffold of controlled porosity. Indeed, the pore nucleation and growth take place with the simultaneous counteract of the vitrification of the matrix, since the plasticization effect of the CO_2_ is reduced with a decrease in pressure. Foaming occurs until the polymer is too stiff to expand, resulting in a solid porous structure. The end scaffold architecture depends on the solubility and diffusivity of the CO_2_ in the polymer, which can be indirectly modulated by modifications of the working parameters (pressure, temperature, and soaking time) (Figure 8b) [82,108]. At a given temperature, the density of CO_2_ increases at higher pressures favoring its solubilization; therefore, a greater amount of CO_2_ is dissolved in the polymer matrix leading to more supersaturation levels upon depressurization [109,110]. An increase in temperature provides both a reduction in CO_2_ density and an increase in diffusivity; consequently, fewer nucleation sites are obtained for scaffolds processed at higher temperatures. In addition, the thermal effect facilitates the chain mobility of the polymer, reducing its viscosity and allowing the pores to grow easier, as well as promoting the pore coalescence phenomena [111,112] (Figure 9a–c). Finally, the soaking time mainly affects the CO_2_ distribution along the polymer, resulting in heterogeneous structures when the soaking time is insufficient to achieve a saturation state [111]. Namely, the increase in the soaking time allows a greater gas dissolution in the polymer, which means more nucleation points that upon the release of pressure would render scaffolds with higher cell densities and reduced pore diameters (Figure 9d–f) [112].

The most accurate equipment to experimentally determine the CO_2_ sorption in a polymer is the magnetic suspension balance, which is a contactless method that is able to weigh the sample under a wide range of pressures and temperatures [98,113].

Supercritical CO_2_ foaming usually produces scaffolds with closed pores and low pore interconnectivity, being key parameters determining both the permeability and cellular infiltration capacity [114]. The use of salt particles and subsequent leaching has been used as a strategy to overcome this limitation [115,116,117]. Other supercritical foaming modifications not requiring downstream processes are under research. Namely, the use of organic solvents [118,119], biofunctional plasticizers [114], or the incorporation of aerogels from different sources [120,121,122,123] in synthetic scaffolds processed by supercritical foaming promoted the formation of more interconnected porous structures with larger pores. The pore interconnectivity can be also modulated by modifications on the venting rate [124]. Uniform porous structures are obtained at slower depressurization rates, allowing the pores to grow and to coalesce, forming open porosities [109,111,125,126].

**Figure 10:**
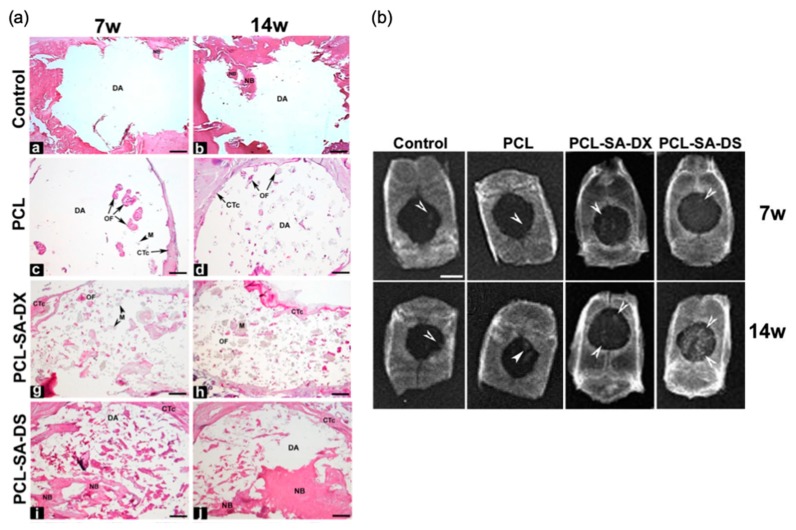
(**a**) Rat crania histological analysis after 7 (7w) and 14 weeks (14w) post-implantation of PCL-based scaffold containing dexamethasone both in the base (DX) and phosphate (DS) forms. Areas of new bone formation are clearly appreciated in PCL-SA-DS formulations. Notation: CTc: connective tissue capsule, DA: defect area, M: material, NB: newly formed bone, OF: ossification foci. (**b**) Accompanying X-ray radiographies of former rat crania. White arrows highlight areas of radiological density compatible with bone neoformation, being remarkably increased for those scaffolds containing dexamethasone. Scale bars: (**a**) 1 mm and (**b**) 4 mm. Figures reprinted from Journal of CO2 Utilization, 31, Goimil, L., Santos-Rosales, V., Delgado, A., Évora, C., Reyes, R., Lozano-Pérez, A. A., Aznar-Cervantes, S. D., Cenis, J. L., Gómez-Amoza, J. L., Concheiro, A., Alvarez-Lorenzo, C., and García-González, C. A., ScCO2-foamed silk fibroin aerogel/poly(ε-caprolactone) scaffolds containing dexamethasone for bone regeneration, 51-64, 2019, with permission from Elsevier. https://doi.org/10.1016/j.jcou.2019.02.016.

The formation of an outer non-porous skin on the scaffolds obtained by supercritical foaming is an intrinsic phenomenon that is also caused by the rapid diffusion of CO_2_ from the surface of the material; thus, the removal of this layer is mandatory before any further use [95]. Although it is a complex dynamic process where many parameters affect the ending structure, supercritical CO_2_ foaming has been established as a versatile and efficient technology for the production of solvent-free scaffolds with remarkable in vivo outcomes [106,127,128]. Namely, supercritical foaming allowed the simultaneous processing of PCL scaffolds coupled with the loading of dexamethasone (up to 5 wt %), showing excellent in vivo compatibility and promoting the bone repair at 14 weeks post-implantation on a critical-sized calvarial defect using a rat model [121] (Figure 10). Furthermore, the bone in-growth in a β-tricalcium phosphate/PLA composite scaffold after 12 months post-implantation in sheep femur and tibia was demonstrated [129].

## 7. Conclusions and Future Trends

The processing of scaffolds for regenerative medicine using solvent-free strategies usually results in advanced materials with attractive morphological, mechanical, and biological properties and with enhanced performance with respect to conventional solvent-based methods. In certain cases, these manufacturing technologies operating in the absence of solvents confer the scaffolds with unique properties. Particularly, the choice of these techniques is clearly advantageous for the processing of medicated scaffolds in terms of loading yield and retained activity. In addition, a significant reduction of the economic burden of the process is achieved when high-cost bioactive agents (e.g., growth factors) are involved. Nevertheless, there are some specific aspects for each of these techniques to be considered as having room for improvement. For instance, a deleterious drug-washing effect typically occurs during post-processing steps (requiring solvents such as leaching) in the melt molding technique. The thermal stability not only of raw materials but also of the employed bioactive agent must be considered in FDM technology, since it could imply a loss of pharmacological activity. Likewise, the processing resolution limitations of the latter technique have to be tackled. In terms of morphology, both sintering techniques and gas foaming present porosity limitations constraining the scaffold design and manufacturing. On the other hand, scaffolds displaying low interconnected and closed pores hampering tissue colonization are usually obtained though compressed CO_2_ foaming. These limitations can be overcome by means of future developments through changes in the formulation (e.g., the use of bifunctional components with dual processing and biological performances [114]) or through a combination of technologies (e.g., the combination of electrospinning technology with FDM technology for an improved porosity control and resolution [130]).

Scaffold processing benefits should also be evaluated from an environmental point of view. Therefore, low carbon footprint and E-factors (i.e., the actual amount of waste produced in the process per gram of product) are two parameters that deserve attention. The absence of solvents, the plasticizing effect of CO_2_ in many polymers, and the valorization of CO_2_ render the compressed CO_2_ foaming technology as particularly attractive. The dramatic decrease in the temperature of thermal events in polymers (mainly T_m_ and T_g_) with this technology is not only of great interest to reduce the heating duties, but also to process medicated scaffolds containing labile ingredients that were unmet before due to the usual limitations of the thermal-based scaffold process.

Finally, current biomedical trends point at the personalized medicine with clinical solutions adapted to each patient. Graft designs that are able to promote controlled and tailor-made release profiles can support this enhanced biological performance. The possibility of getting a CT or MRI image of the tissue defect from the patient combined with the option to produce unique personalized formats by some solvent-free approaches (e.g., FDM and SLS technologies) is very attractive and needs to be further explored, especially with medicated scaffolds.
